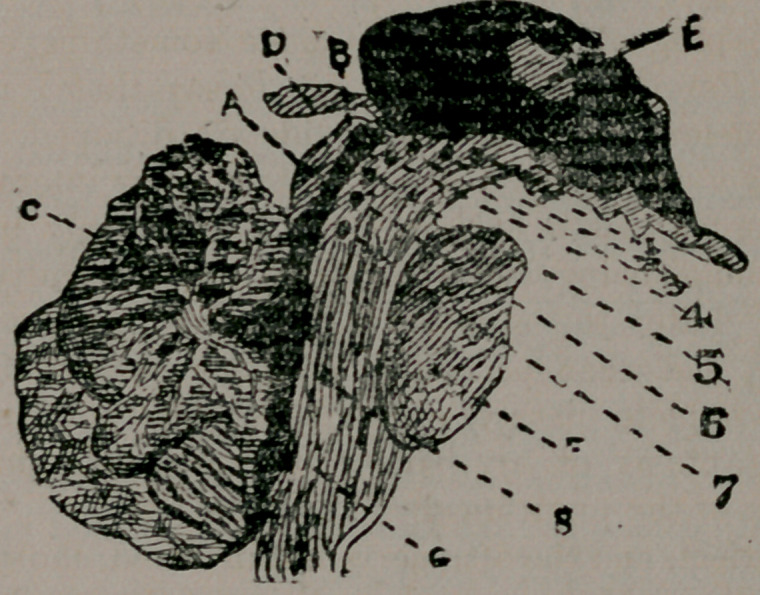# A Case of Ptosis and Ophthalmoplegia Exterior

**Published:** 1890-01

**Authors:** W. L. Bullard

**Affiliations:** Columbus, Ga.


### A CASE OF PTOSIS AND OPHTHALMOPLEGIA EXTERIOR.

BY W. L. BULLARD, M. D., COLUMBUS, GA.

The above cut is from a photo of Master J. W., æt. 17, Ellaville, Ga. His trouble commenced over seven years ago. He is small for one of his age, weight eighty pounds, with feminine look and voice. Dr. C. H. Smith, the family physician, writes me that the mother has attacks of asthma, otherwise the family history is good.

Now when the novice looks at the cut he may remark as did a practicing physician and special friend to whom I showed the photograph: "Yes, I have seen quite a number of cases like this." I quickly asked would he be so kind as to give me his experience with this class of cases, as I had seen only two others—one when at Moonfields, London, and one in the private office of my friend, Dr. W. F. Mittendorf, of New York city. "Well," said he, "I see that I am mistaken; there is some other trouble besides ptosis."

So drooping of the lids may mean something else besides ptosis. (Parenthetically allow me to say that I now have a patient under treatment whose lids are drooped, pupils contracted, &c. Horner's disease—to me a very interesting case.) All of the muscles in each eye have successively become paralyzed, commencing first with ptosis and a difficulty in looking upward. Later the eyes moved in all directions with great difficulty, and when patient came under my care there existed almost complete paralysis of all the external muscles, with slight praptosis of eye-balls; the accommodation and the sphincter of the pupil unaffected with vision. * * * Vision being perfect, and the fifth nerve not involved, shows the lesion to be central, and the normal condition of the pupil and accommodation is proof that it is not the trunk of the *moto oculi*, but the nuclei of the external musles that are affected, which is confirmatory as to the special localization of the nuclei lately established by Pick, Kahlar, Hensen and Volckers. The lesion, it is said, is located in the nuclei beneath the aqueduct of sylvius, and in the floor of the fourth ventricle.

Bery and Branwell (1) report a case with recovery. The lesion they trace to tubercle at the top of the pons, near the aqueduct of sylvius.

Kojewnikoff (2) reports a case which ended fatally. The autopsy showed capillary hemorrhage, with softening of the gray matter limited to the floor of the fourth ventricle, the confines of the nucleus of the third nerve, and aqueduct of sylvius extending symmetrically upon two sides.

This case is reported on account of its rarity. I don't remember ever having seen the report of a case from the South. My patient has been under treatment two months, and while the movements of the balls in all direction, some upwards, are enlarged, the improvement is not at all rapid.

In connection with internal medication, I am using electricity as designated by Dr. Buggord, namely, for the surgeon to place himself in the circuit, and use his index finger as a reophore. The accompanying diagram shows the relation of the nuclei.

(1) Edinburg Medical Journal.
(2) Annual Universal Medical Sciences.

—from *Medical Progress.*

Longitudinal Ventrical Section through the human brain, showing (diagrametrically) the nerve-nuclei of the ocular muscles. A. Testes, and B notes of corpora quadrigermina. C. Cerebellum. D. Pineas gland. E. Soft commissure in the middle of the third ventricle, which, with the aqueduct of sylvius and fourth ventricle, are represented in block. F. The protuberance of the pons. G. Medulla oblongata. 1 to 6 different parts of the third nerve-nucleus, viz.: 1. Centre for accommodation. 2. Centre for sphincter of the pupil. 3. Centre for internal rectus. 4. Centre for the rectus superior. 5. Centre for the levator selpebræ luperioris. 6. Centre for the rectus superior. 7. Centre of the trochlearis for the superior oblique. 8. Centre of the sixth nerve for the external rectus. —*Edinburgh Med. Jour.*—from annual of the *Universal Medical Sciences*, 1888.

---

Chas. Chadwick, Ottis R. Wyeth, Louis A. Schoen, George J. Schoen, Charles F. Herman, George Eyesell and Horace L. Roy, druggists, of Kansas City, Mo., were recently fined $500 each and costs for counterfeiting a preparation known as Bromidia.—*Journal of the American Medical Association*, Chicago, November 16, 1889.

## Figures and Tables

**Figure f1:**
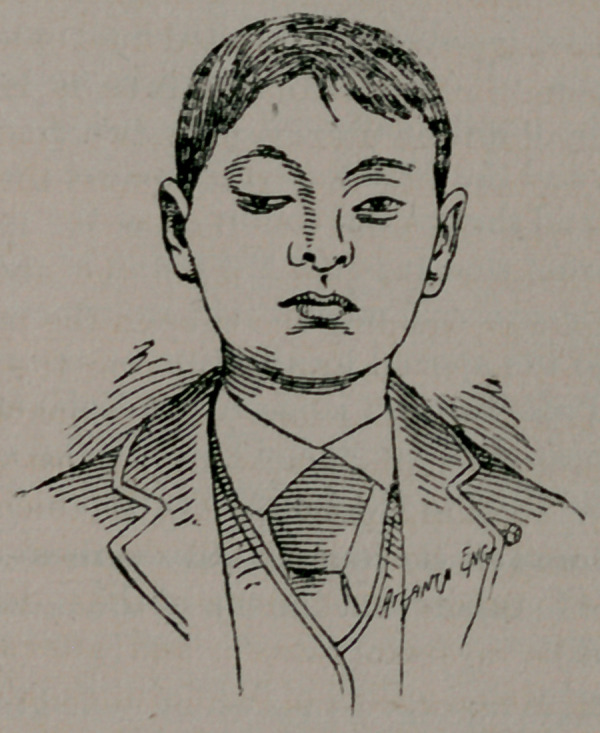


**Figure f2:**